# IgG4-Related Disease: A Case Series Highlighting Diverse Clinical Manifestations and Treatment Outcomes

**DOI:** 10.7759/cureus.94790

**Published:** 2025-10-17

**Authors:** Thanda Aung, Kaitlin Eblen, Gregory A Fishbein

**Affiliations:** 1 Rheumatology, University of California Los Angeles David Geffen School of Medicine, Los Angeles, USA; 2 Pathology and Laboratory Medicine, University of California Los Angeles David Geffen School of Medicine, Los Angeles, USA

**Keywords:** igg4-related disease, lymphadenopathy, mikulicz's disease, sclerosing mesenteritis, sicca syndrome

## Abstract

IgG4-related disease (IgG4-RD) is a systemic immune-mediated fibroinflammatory condition characterized by tumor-like swelling of affected organs, lymphoplasmacytic infiltration enriched with IgG4-positive plasma cells, and variable degrees of fibrosis. We present three cases of IgG4-RD with diverse clinical presentations, highlighting the diagnostic challenges and management approaches. Our cases include a 45-year-old female with lacrimal and salivary gland involvement mimicking Sjögren's syndrome, a 77-year-old male with pancreatic mass, mesenteric vasculitis, and intra-abdominal lymphadenopathy initially suspected as pancreatic cancer, and a 75-year-old male with diffuse lymphadenopathy, initially thought to be lymphoma. All patients were successfully treated with rituximab with good clinical and radiological responses, emphasizing the importance of considering IgG4-RD in the differential diagnosis of conditions with multisystem involvement and atypical presentations.

## Introduction

IgG4-related disease (IgG4-RD) is a recently recognized immune-mediated condition characterized by tumefactive lesions, dense lymphoplasmacytic infiltrate rich in IgG4-positive plasma cells, storiform fibrosis, and often elevated serum IgG4 levels [1]. First described in association with autoimmune pancreatitis in 2001, IgG4-RD is now recognized as a systemic disease affecting virtually any organ system [2,3].

The clinical presentation of IgG4-RD is highly variable, frequently leading to misdiagnosis or delayed diagnosis. The disease commonly mimics malignancies, infectious processes, or other inflammatory conditions, making histopathological confirmation essential for definitive diagnosis [4]. Key histopathological features include lymphoplasmacytic infiltration with IgG4-positive plasma cells (>10 cells/high-power field and/or IgG4+/IgG+ plasma cell ratio >40%), storiform fibrosis, and obliterative phlebitis [1].

While glucocorticoids remain first-line therapy, rituximab has emerged as an effective steroid-sparing agent for refractory disease [5]. Rituximab, a monoclonal antibody against CD20-positive B cells, demonstrates efficacy in inducing and maintaining remission by depleting precursors of IgG4-producing plasma cells [6].

We present three cases of IgG4-RD with markedly different clinical presentations, emphasizing the diagnostic challenges and therapeutic considerations in this complex disease entity.

## Case presentation

Case 1: IgG4-related ophthalmic disease with sicca symptoms

A 45-year-old female presented with progressive enlargement of the right lacrimal gland, worsening over seven months. She initially experienced eyelid swelling and hypoglobus, which temporarily improved with a one-month course of oral prednisone (40 mg tapered over four weeks) but recurred one week after completing treatment.

**Figure 1:**
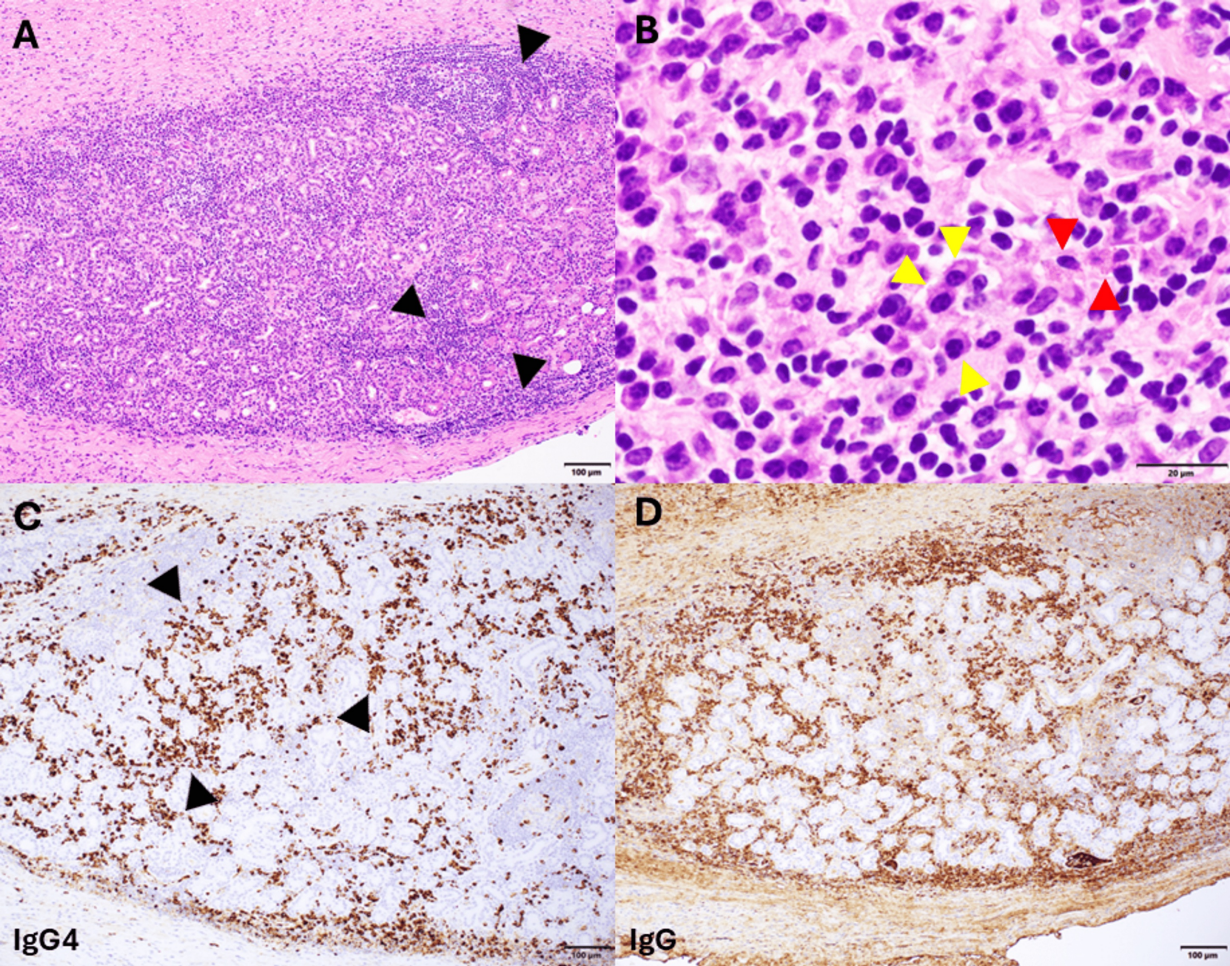
IgG4-related ophthalmic disease. (A) Low power (100x) hematoxylin and eosin (H&E)-stained section of lacrimal gland with abundant lymphoplasmacytic inflammation and a background of fibrosis (black arrows). (B) High-power (600x) H&E showing numerous plasma cells (yellow arrows) accompanied by scattered eosinophils (red arrows). (C) 100x field demonstrating IgG4-positive plasma cells (black arrows pointing at brown cells) by immunohistochemistry: up to ~200 per high power field. (D) IgG IHC demonstrating that the IgG4-positive cells represent at least 40% of the IgG-positive plasma cells.

Physical examination revealed asymmetrically enlarged lacrimal glands, particularly the right side, and submandibular lymphadenopathy. She reported sicca symptoms, including dry eyes and mouth. Serum IgG4 levels were persistently elevated (ranging from 174-231 mg/dL). Serological evaluations for Sjögren’s syndrome, systemic lupus erythematosus, and sarcoidosis were negative, including negative results for antinuclear antibody (ANA), anti-anti-double-stranded DNA (anti-dsDNA), anti-Sjögren’s-syndrome-related antigen A (anti-SSA), anti-Sjögren’s-syndrome-related antigen B (anti-SSB), and antineutrophil cytoplasmic antibody (ANCA). Excisional biopsy of the right lacrimal gland revealed prominent follicular lymphoid hyperplasia, dense perifollicular lymphoplasmacytic infiltrate, and marked fibrosis in a focal storiform pattern. Immunohistochemical studies demonstrated increased IgG4-positive plasma cells (IgG4/IgG >40%, IgG4 >100 per HPF overall, and >200 per HPF in some areas), confirming the diagnosis of IgG4-related ophthalmic disease (Figure 1).

**Figure 2:**
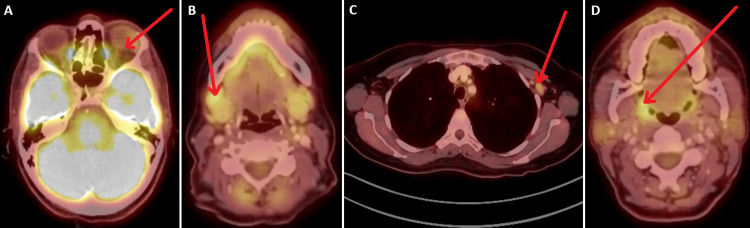
PET/CT showing FDG uptake in the left lacrimal gland, right submandibular lymph node, left axillary lymph node, and right tonsilar area. (A) Demonstration of a thickened left lacrimal gland with moderate fluorodeoxyglucose (FDG) activity (red arrow). (B) Right submandibular lymph node with intense FDG uptake (red arrow). (C) Left axillary lymph node with moderate FDG activity (red arrow). (D) Right tonsillar FDG uptake (red arrow).

PET/CT imaging showed FDG-avid lesions in the left lacrimal gland, right submandibular lymph node, left axillary lymphadenopathy, and asymmetric tonsillar activity (Figure 2). After initial treatment with prednisone and a period of observation, the patient received rituximab (1,000 mg given twice, two weeks apart) in June 2024. Follow-up evaluation four weeks later showed excellent response with resolution of lacrimal and submandibular gland swelling, normalization of erythrocyte sedimentation rate (ESR), and IgG levels. PET/CT in December 2024 demonstrated resolution of previously noted hypermetabolic activities. As of April 2025, the patient showed a slight elevation of CD19 (4 cells/μL) but remained asymptomatic with overall improvement on imaging.

Case 2: IgG4-related sclerosing mesenteritis with pancreatic involvement

A 77-year-old male presented with mesenteric and retroperitoneal lymphadenopathy, chylous ascites requiring paracentesis, and a pancreatic mass initially suspected to be malignant. The patient reported abdominal discomfort and increasing abdominal girth. Physical examination revealed ascites and abdominal tenderness.

**Figure 3:**
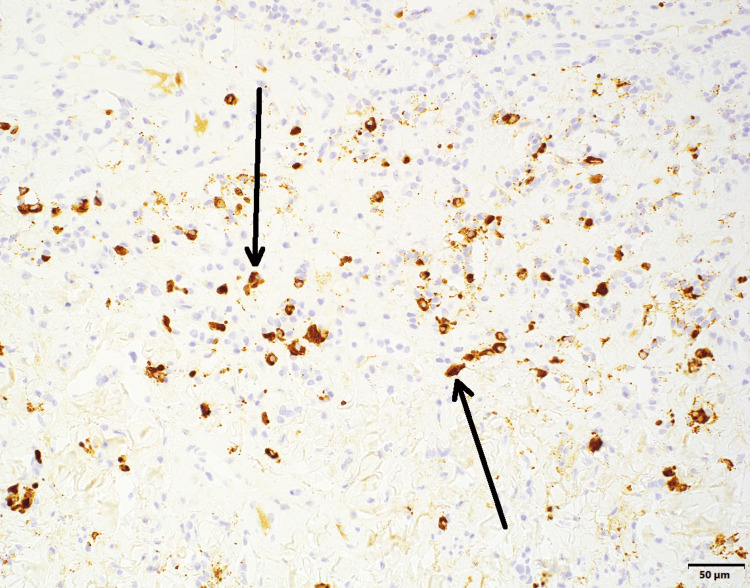
Immunohistochemical stain for immunoglobulin G4 (IgG4) with hematoxylin counterstain in mesenteric biopsy Black arrows pointing at IgG4-positive plasma cells.

**Figure 4:**
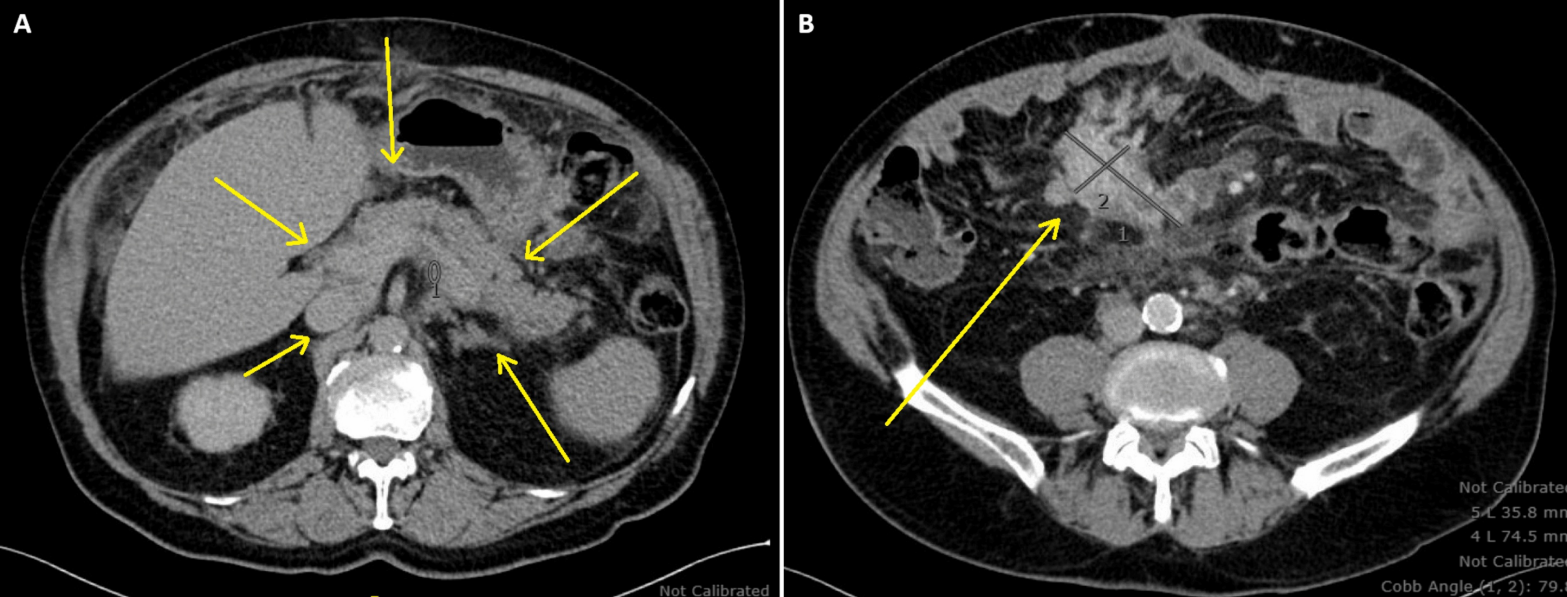
Abdominal computed tomography (CT). (A) Homogeneously enhancing, lobulated pancreatic parenchyma (yellow arrows). (B) Enhancing, spiculated soft-tissue lesion in the right central mesentery, suggestive of sclerosing mesenteritis (yellow arrow).

Mesenteric biopsy in January 2023 showed sclerosing mesenteritis with increased IgG4-positive plasma cells consistent with IgG4-related disease (Figure 3). The patient was referred to an outside oncologist due to initial concerns for malignancy. Serum immunoglobulin studies revealed normal IgG4 levels (37-54 mg/dL) throughout the disease course, but significantly elevated IgG1 levels (933-998 mg/dL) and occasionally elevated IgG2 levels (162-179 mg/dL). Imaging studies demonstrated an infiltrative retroperitoneal and mesenteric process surrounding the pancreatic head/neck, mesenteric root, and porta hepatis, with mesenteric adenopathy and peritoneal/omental stranding with ascites (Figure 4).

Extensive workup ruled out malignancy, with negative ascitic fluid analysis for malignant cells and AFB. Liver scans showed no evidence of cirrhosis. The patient was initially treated with prednisone 20 mg daily with limited response. In June 2023, rituximab therapy was initiated (1000 mg given twice, two weeks apart), followed by azathioprine 150 mg daily and a prednisone taper. This regimen led to clinical improvement with decreased ascites, abdominal girth reduction (from 48.5 to 46.5 inches), and gradual normalization of IgG1 levels from the 900s to 684-702 mg/dL by 2025. Due to elevations in liver enzymes, azathioprine was discontinued.

The patient received subsequent cycles of rituximab in December 2023, July 2024, and January/February 2025. PET/CT in December 2024 showed resolution of ascites and improvement in retroperitoneal and mesenteric disease. As of February 2025, the patient had successfully discontinued prednisone and reported only intermittent sensations of abdominal pressure with decreasing abdominal girth.

Case 3: IgG4-related lymphadenopathy

A 75-year-old male with a history of hypertension, hypothyroidism, hyperlipidemia, and chronic hepatitis B infection presented with worsening lymphadenopathy since November-December 2018. The patient had generalized lymphadenopathy without localizing symptoms.

**Figure 5:**
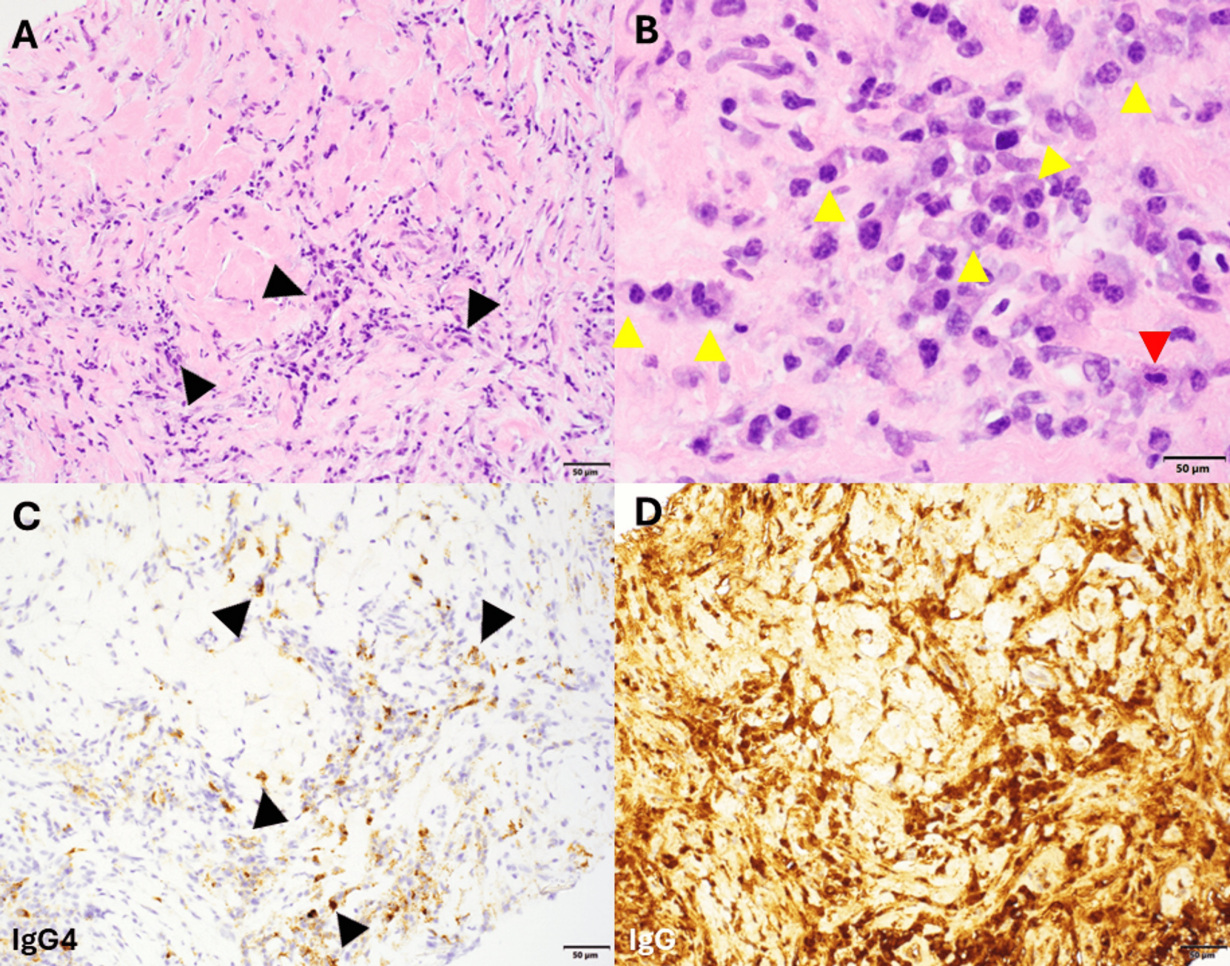
IgG4-related lymphadenopathy. (A) Hematoxylin and eosin (H&E)-stained section (200x) showing a sclerotic lymph node with lymphoplasmacytic infiltrate (black arrows). (B) Numerous plasma (yellow arrows) cells and occasional eosinophils (red arrow) are seen (scale bar = 50 microns). (C) IgG4 immunohistochemistry (IHC) demonstrates increased IgG4-positive plasma cells (black arrows pointing at brown cells) (200x). (D) IgG IHC demonstrating that the IgG4-positive plasma cells represent roughly 40% of the total number of plasma cells (200x).

Initial lymph axillary node biopsies in April 2019 showed acute lymphadenitis. A subsequent right axillary lymph node biopsy in July 2019 revealed dense fibrosis with acute and chronic inflammation, including numerous neutrophils and plasma cells, with increased IgG4-positive plasma cells (up to 40/HPF) (Figure 5).

**Figure 6:**
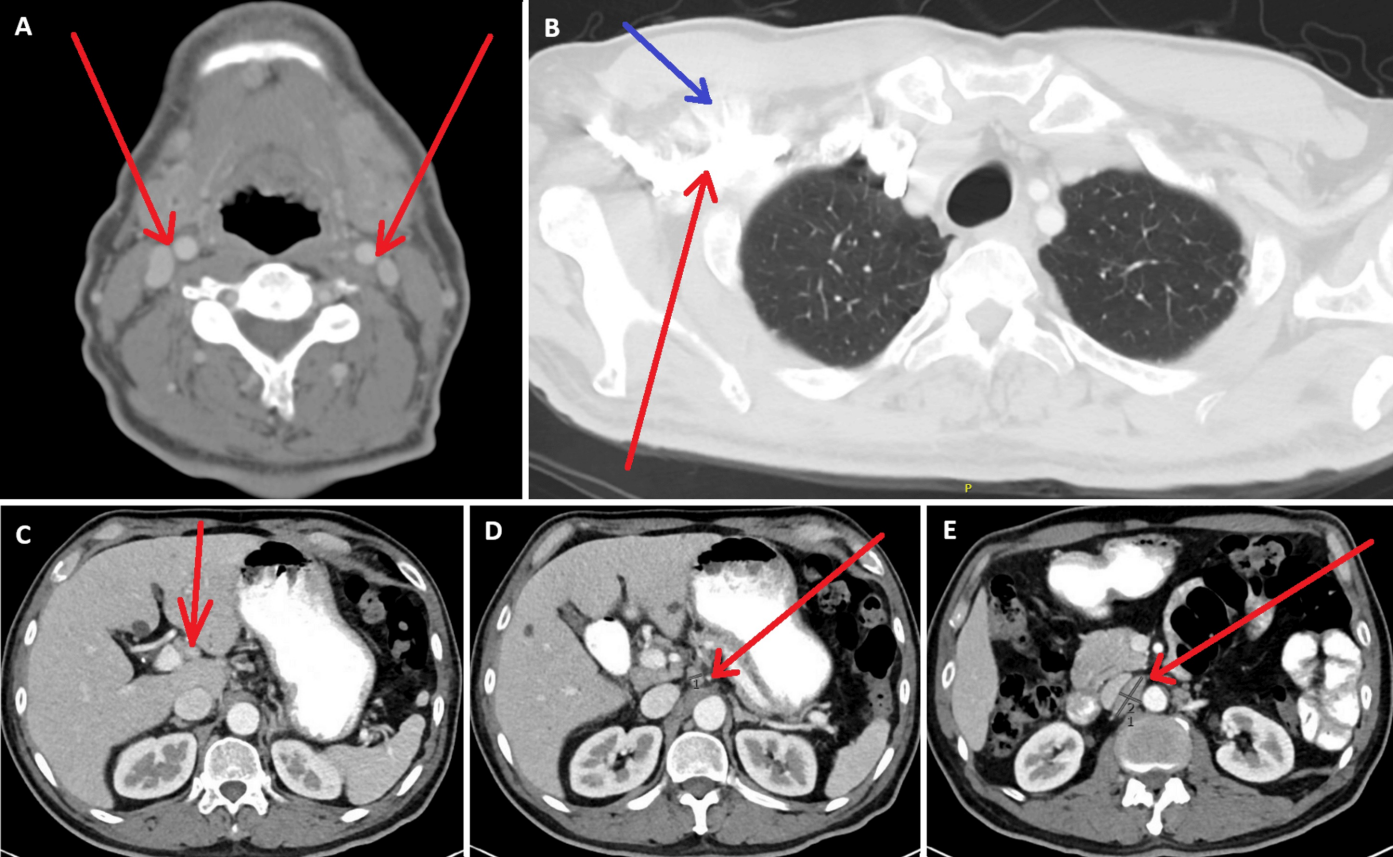
CT imaging of the neck, chest, and abdomen demonstrating multistation lymphadenopathy. (A) Neck CT demonstrating bilateral upper cervical chain adenopathy (red arrows). (B) Chest CT demonstrating several prominent and enlarged right axillary lymph nodes (red arrow) with adjacent fat stranding (blue arrow). (C-E) CT abdomen demonstrating multistation retroperitoneal and abdominal lymphadenopathy (red arrows), including an enlarged periportal lymph node (C), gastroduodenal and gastrohepatic lymph nodes (D), and bulky right para-aortic lymphadenopathy (E).

Notably, serum IgG4 levels were consistently within normal limits (ranging from 28-70 mg/dL) throughout the disease course, but the patient had markedly elevated IgG1 levels (1,214 mg/dL) at presentation in early 2023. The patient also had elevated inflammatory markers with an ESR of 86 mm/hour. Extensive workup ruled out infectious etiologies, including fungal, bacterial, and mycobacterial infections, although tuberculosis testing was indeterminate. CT imaging showed multistation lymphadenopathy (Figure 6).

The patient was started on prednisone 40 mg daily in September 2019 with significant improvement in lymph node burden and a decrease in inflammatory markers (ESR 86→75→41→normal). Rituximab therapy was initiated in November 2019, with subsequent cycles administered every six months, then extended to every nine months. CT imaging in March 2021 showed resolution of lymphadenopathy.

With rituximab treatment, IgG1 levels progressively normalized from the initial 1,214 to 702 mg/dL by 2025. The patient continued with rituximab maintenance therapy, with monitoring of IgG subclasses. In 2024, the rituximab schedule was adjusted back to every six months. By January 2025, the patient was doing well, had discontinued prednisone, and maintained remission with no evidence of lymphadenopathy (Tables 1-2).

**Table 1:** IgG subclasses level pre- and post-treatment in three cases. IgG, immunoglobulin G

	Normal reference range	Case 1		Case 2		Case 3	
		Pre-treatment	Post-treatment	Pre-treatment	Post-treatment	Pre-treatment	Post-treatment
IgG1	240-1,118 mg/dL	Normal	Normal	1,379	674	1,470	753
IgG2	124-549 mg/dL	Normal	Normal	Normal	Normal	Normal	Normal
IgG3	21-134 mg/dL	Normal	Normal	Normal	Normal	Normal	Normal
IgG4	1-123 mg/dL	231	57	Normal	Normal	Normal	Normal

**Table 2:** Inflammation markers in three cases, pre- and post-treatment. ESR, erythrocyte sedimentation rate; CRP, C-reactive protein

		Normal reference range	Pre-treatment	Post-treatment
Case 1	ESR	<=25 mm/hour	43 mm/hour	9 mm/hour
	CRP	<0.8 mg/dL	1.1 mg/dL	<0.3 mg/dL
Case 2	ESR	<=25 mm/hour	90 mm/hour	21 mm/hour
	CRP	<0.8 mg/dL	4.7 mg/dL	<0.3 mg/dL
Case 3	ESR	<=25 mm/hour	65 mm/hour	13 mm/hour
	CRP	<0.8 mg/dL	2.5 mg/dL	<0.3 mg/dL

## Discussion

Clinical heterogeneity and diagnostic challenges

Our case series exemplifies the protean manifestations of IgG4-RD, demonstrating presentations mimicking Sjögren's syndrome, pancreatic malignancy, and lymphoma. This clinical heterogeneity underscores the importance of maintaining high diagnostic suspicion in patients with unexplained multiorgan involvement [7].

Serological considerations

A critical finding from our series is that normal serum IgG4 levels do not exclude IgG4-RD diagnosis. Two of three patients maintained normal IgG4 levels despite biopsy-proven disease, while demonstrating significant IgG1 elevations. This observation aligns with recent literature emphasizing that serum IgG4 alone is insufficient for diagnosis or exclusion of IgG4-RD [8]. The elevation of alternative IgG subclasses, particularly IgG1, represents an important atypical serological pattern that clinicians should recognize.

Histopathological confirmation

All cases demonstrate the critical importance of tissue diagnosis. The histopathological hallmarks - lymphoplasmacytic infiltration with IgG4-positive plasma cells, storiform fibrosis, and obliterative phlebitis - were evident despite variable serological presentations [4]. This reinforces the 2019 American College of Rheumatology (ACR)/European Alliance of Associations for Rheumatology (EULAR) classification criteria, emphasizing histopathological features for a definitive diagnosis [9].

Therapeutic considerations

Glucocorticoid Limitations

While glucocorticoids achieve initial remission in most patients, our cases illustrate their limitations, including partial responses and high relapse rates upon discontinuation [1]. Additionally, prolonged use carries substantial risks, particularly in elderly patients [3].

Inebilizumab Efficacy

According to Stone et al., inebilizumab reduced the risk of flare-ups in IgG4-related disease and significantly increased the likelihood of achieving complete, flare-free remission after one year [10]. These findings support the potential of CD19-targeted B-cell depletion as an effective treatment strategy for IgG4-related disease. Inebilizumab was approved by the U.S. Food and Drug Administration (FDA) for this indication in April 2025 [11].

Rituximab Efficacy

All three patients demonstrated excellent responses to rituximab, consistent with reported response rates in refractory disease [5,6]. The mechanism involves B-cell depletion, targeting precursors of IgG4-producing plasma cells and attenuating fibrosis biomarkers [7,12]. Our experience supports maintenance rituximab therapy with individualized dosing intervals based on clinical and serological parameters [13].

Combination and Alternative Therapies

Case 2 illustrates potential benefits of rituximab-azathioprine combination therapy, though this must be balanced against increased adverse effect risks [12]. Other agents, including methotrexate, mycophenolate mofetil, and emerging biologics, may have roles in selected patients [1,3].

Age-related considerations

Two patients (aged 75 and 77 years) highlight challenges in elderly patient management, requiring careful risk-benefit assessment for aggressive immunosuppression. Our cases demonstrate that age should not preclude effective treatment, as both elderly patients achieved sustained remission.

Long-term monitoring and outcomes

Regular monitoring through clinical examination, inflammatory markers, immunoglobulin levels, and imaging proved essential for treatment optimization [14]. The ability to extend rituximab intervals in sustained remission (Case 3) while shortening them for subclinical activity suggests that individualized maintenance approaches are preferable to fixed protocols.

Limitations

Our case series is limited by a small sample size and retrospective analysis. The heterogeneous presentations, while illustrative of disease diversity, may not represent typical cases. Additionally, long-term follow-up data remain limited for assessing treatment durability and late complications.

## Conclusions

IgG4-RD presents diagnostic challenges due to diverse clinical manifestations, as illustrated by our three cases involving lacrimal/salivary glands, pancreatic/mesenteric tissue, and diffuse lymphadenopathy. Importantly, normal serum IgG4 levels do not exclude the diagnosis, as two patients maintained normal IgG4 with elevated IgG1, emphasizing the critical role of histopathological confirmation over serology alone. Rituximab proved effective in all cases, achieving clinical, radiological, and laboratory improvements with individualized treatment intervals essential for maintaining remission. A high index of suspicion and multidisciplinary collaboration remain crucial for the diagnosis and management of this increasingly recognized condition. Standardized diagnostic criteria and evidence-based treatment algorithms are needed to optimize patient care.
